# A scalable hyperthermic intravesical chemotherapy (HIVEC) setup for rat models of bladder cancer

**DOI:** 10.1038/s41598-022-11016-y

**Published:** 2022-04-29

**Authors:** J. W. Van Hattum, E. M. Scutigliani, R. F. C. P. A. Helderman, R. Zweije, H. M. Rodermond, A. L. Oei, J. Crezee, J. R. Oddens, T. M. De Reijke, P. M. Krawczyk

**Affiliations:** 1 Department of Urology, Amsterdam University Medical Centers, University of Amsterdam, Meibergdreef 15, 1105 AZ Amsterdam, The Netherlands; 2 Department of Medical Biology, Amsterdam University Medical Centers, Cancer Center Amsterdam, University of Amsterdam, Meibergdreef 15, 1105 AZ Amsterdam, The Netherlands; 3 Laboratory for Experimental Oncology and Radiobiology (LEXOR), Center for Experimental and Molecular Medicine (CEMM), Amsterdam University Medical Centers, Cancer Center Amsterdam, University of Amsterdam, P.O. Box 22700, 1100 DE Amsterdam, The Netherlands; 4 Department of Radiation Oncology, Amsterdam University Medical Centers, Cancer Center Amsterdam, University of Amsterdam, P.O. Box 22700, 1100 DE Amsterdam, The Netherlands

**Keywords:** Bladder cancer, Preclinical research

## Abstract

Hyperthermic intravesical chemotherapy (HIVEC)—whereby the bladder is heated to ± 43 °C during a chemotherapy instillation—can improve outcomes of non-muscle invasive bladder cancer (NMIBC) treatments. Experiments in animal models are required to explore new hyperthermia based treatments. Existing HIVEC devices are not suitable for rodents or large-scale animal trials. We present a HIVEC setup compatible with orthotopic rat models*.* An externally heated chemotherapeutic solution is circulated in the bladder through a double-lumen catheter with flow rates controlled using a peristaltic pump. Temperature sensors in the inflow channel, bladder and outflow channel allow temperature monitoring and adjustments in real-time. At a constant flow rate of 2.5 mL/min the system rapidly reaches the desired bladder temperature of 42–43 °C with minimal variability throughout a one-hour treatment in a rat bladder phantom, as well as in euthanised and live rats. Mean intraluminal bladder temperatures were 42.92 °C (SD = 0.15 °C), 42.45 °C (SD = 0.37 °C) and 42.52 °C (SD = 0.09 °C) in the bladder phantom, euthanised, and live rats respectively. Thermal camera measurements showed homogenous heat distributions over the bladder wall. The setup provides well-controlled thermal dose and the upscaling needed for performing large scale HIVEC experiments in rats.

## Introduction

With almost 550,000 new confirmed cases in 2018 worldwide, bladder cancer (BC) is the 6th and 17th most frequently diagnosed malignancy in men and women, respectively^1^. The majority (75–85%) of patients present with non-muscle-invasive bladder cancer (NMIBC)^2^. Although NMIBC has a favourable 5-year survival rate of 70%, this is offset by a high 5-year recurrence rate (50–70%). Moreover, there is a 10–20% chance of progression to muscle-invasive bladder cancer (MIBC), which reduces the 5-year survival rate up to 50%^3,4^. The standard treatment for NMIBC consists of transurethral resection of the entire visible tumour (TUR-T), followed by intravesical instillations with either the immunostimulatory Bacillus Calmette–Guérin (BCG) or chemotherapy (e.g. mitomycin-C or epirubicin) . Depending on the clinicopathological risk group for recurrence and progression, the number of instillations varies from a single dose of chemotherapy, immediately following TUR-T in the low-risk category, to a series of repeated instillations for up to one year with chemotherapeutics, or one to three years with BCG in the intermediate- and high-risk groups^2^. A radical cystectomy (RC) or chemoradiotherapy is recommended for NMIBC patients in the highest risk group and those with MIBC. However, not all patients are suitable or willing to undergo RC or chemoradiotherapy due to high rates of morbidity, mortality, and a negative impact on quality of life^5–7^. Therefore, alternative treatment regimens that prevent the recurrence of NMIBC, and minimize its chance to progress to MIBC, are urgently needed to improve the outcomes.

One such regimen is hyperthermic intravesical chemotherapy (HIVEC), in which chemotherapeutic solutions are introduced into the bladder, while maintaining the bladder wall at supra-physiological—i.e. hyperthermic—temperatures of 42–43 °C for approximately one hour. To achieve the desired temperature, most methods use either radiofrequency waves^8^, applied with intravesical or external antennas, or external heating of the chemotherapeutic solution that is then circulated into and out of the bladder, through a double-lumen catheter^9^.

Hyperthermia exerts various microscopic and macroscopic effects that enhance the efficacy of multiple other treatments, the most established being increased perfusion of the tumour, which enhances drug delivery into various tumour types, including bladder cancer^10–12^. At a molecular level, hyperthermia triggers large-scale protein unfolding, affecting a wide range of cellular processes^13,14^, of which the interference with DNA repair mechanisms plays an important role in the promotion of hyperthermia-based treatment regimens^15^. In addition, various in vitro studies have demonstrated that heat alters membrane characteristics and permeability, likely increasing intracellular concentrations of chemotherapeutic agents^16–19^. In line with these observations, increased cytotoxicity of various chemotherapeutics in combination with hyperthermia has been observed in bladder- and a variety of cancer cell lines^20–22^. In vivo animal studies show that HIVEC increases the concentration of mitomycin-C in the bladder wall, and inhibits tumour growth, compared to treatment at room temperature^23–26^. In line with these findings, clinical evidence further supports the potential of HIVEC. Although currently HIVEC is only considered as a treatment alternative for patients after BCG-failure who cannot undergo radical cystectomy^2^, a growing body of evidence demonstrates that HIVEC can reduce tumour recurrence rates, compared with standard therapy, as well as produce promising long term bladder preservation rates after BCG-failure^27–30^. These studies underscore the potential of HIVEC but also emphasize the need to further explore its mechanisms of action, efficacy and applicability in preclinical investigations, using reliable experimental systems.

Multiple rat models have been established to study bladder cancer biology and treatment response in vivo^31^. Orthotopic cancer models closely resemble canonical bladder cancer development^32^ and thus appear to be more suitable than heterotopic models for recapitulating the clinical HIVEC treatments. There are several methods to simulate HIVEC in animal models, such as conductive heating^25,33,34^, radiofrequency induced hyperthermia (RF-HT)^35–38^, magnetic nanoparticles^26,39^, photothermal ablative therapy^40,41^, and high intensity focused ultrasound (HIFU)^24,42^. These techniques vary in technical complexity and compatibility with orthotopic tumour models (Table 1). Published in vivo studies focusing on local heating of the bladder generally rely on large animals (e.g. rabbit, pig, sheep) as model systems, in conjunction with clinically used HIVEC systems, or large experimental setups unsuitable for rats^12,23,38,43^. On the other hand, systems for local heating of the bladder in rats based on capacitive heating techniques or magnetic fluid hyperthermia^37,39,44^, pose high technical demands that make them challenging to implement and are thus incompatible with larger scale experiments. To our knowledge, a practical and scalable HIVEC system using conductive heating for rats with orthotopic bladder tumour models has not yet been developed^45^.

**Table 1:** Overview of several methods to simulate HIVEC in animal models.

Technique	Location	Animal	Technical specification	Tumour model tested (compatibility)	Technical Complexity	Example
Radiofrequency induced hyperthermia	Intravesical	Pig/Sheep	Clinically used 915 MHz intravesical antenna	None (orthotopic)	Low (Catheter not suited for smaller animals)	van Valenberg et al.^35^ Rath-Wolfson et al.^38^
	Regional pelvic	Mice	2.45 GHz external applicator	None (both)	High	Salahi et al.^37^
	Regional pelvic	Rat	434 MHz external applicator	None (both)	High	Haveman et al.^36^
Conductive heat	Regional extremity	Athymic mice	Submersion of the hind leg in a thermostatically controlled water bath	Heterotopic subcutaneously hind leg	Low	Amano et al.^25^
	Intraperitoneal injection	Mice	Single preheated intraperitoneal injection	Heterotopic in abdomen	Low	Orsolic et al.^33^
	Intravesical closed recirculation system	Pig	Custom made circulatory system connected to a transurethral catheter	None (orthotopic)	Low (Catheter not suited for smaller animals)	Mikhail et al.^12^
	Intravesical injection	Rabbit	Repeated injection every 3 min via transurethral catheter	None (orthotopic)	Low	Ucar et al. ^34^
Magnetic nanoparticles	Intravesical	Rats	Magnetic field applicator (Actium Biosystems, Boulder, CO), 40 kHz, strength to 6 kA/m	None (orthotopic)	High	Oliveira et al.^39^
	Intratumoural injection	Mice	Exposure of intratumourally applied nanoparticles to an alternating magnetic field	Heterotopic subcutaneously	High	Stapf et al. ^26^
Photothermal ablative therapy	Intravesical	Mice	Intravesical gold nanoparticles instillation treated with externally administered 808 nm diode laser	Orthotopic	High	Yang et al. ^40^
	Intravesical Single-walled carbon nanohorns delivered with fiberoptic microneedle	Pig ex vivo	laser heating of infused SWNHs in the bladder wall using a 1,064 nm CW diode-pumped fibre laser (IPG Photonics, Oxford, MA)	None (both)	High	Hood et al.^41^
Ultrasound	Pelvic Magnetic resonance-guided high-intensity focused ultrasound (MRgHIFU)	Pig	256-channel phased-array transducer with a radius of curvature of 70 mm. At a frequency of 1 MHz and pressure of − 6 dB	None (both)	High	Zhu et al. ^42^
	Regional ultrasonic hyperthermia	Rat	2.25 MHz piezoelectric ceramic disc transmitting acoustic wave	Heterotopic Subcutaneously	High	Longo et al.^24^

Here, we present a dedicated bladder instillation system, based on a double-lumen catheter, that is suitable for performing chemohyperthermia (CHT) in rats. We demonstrate that the system can achieve well-controlled local heating of the bladder that can be reliably monitored, maintained and adjusted during treatment. Importantly, it is cost-effective and scalable, making it suitable for preclinical studies in different animal models to accelerate the improvement of existing and development of novel intravesical bladder cancer treatments.

## Material and Methods

### Global setup overview

**Figure 1:**
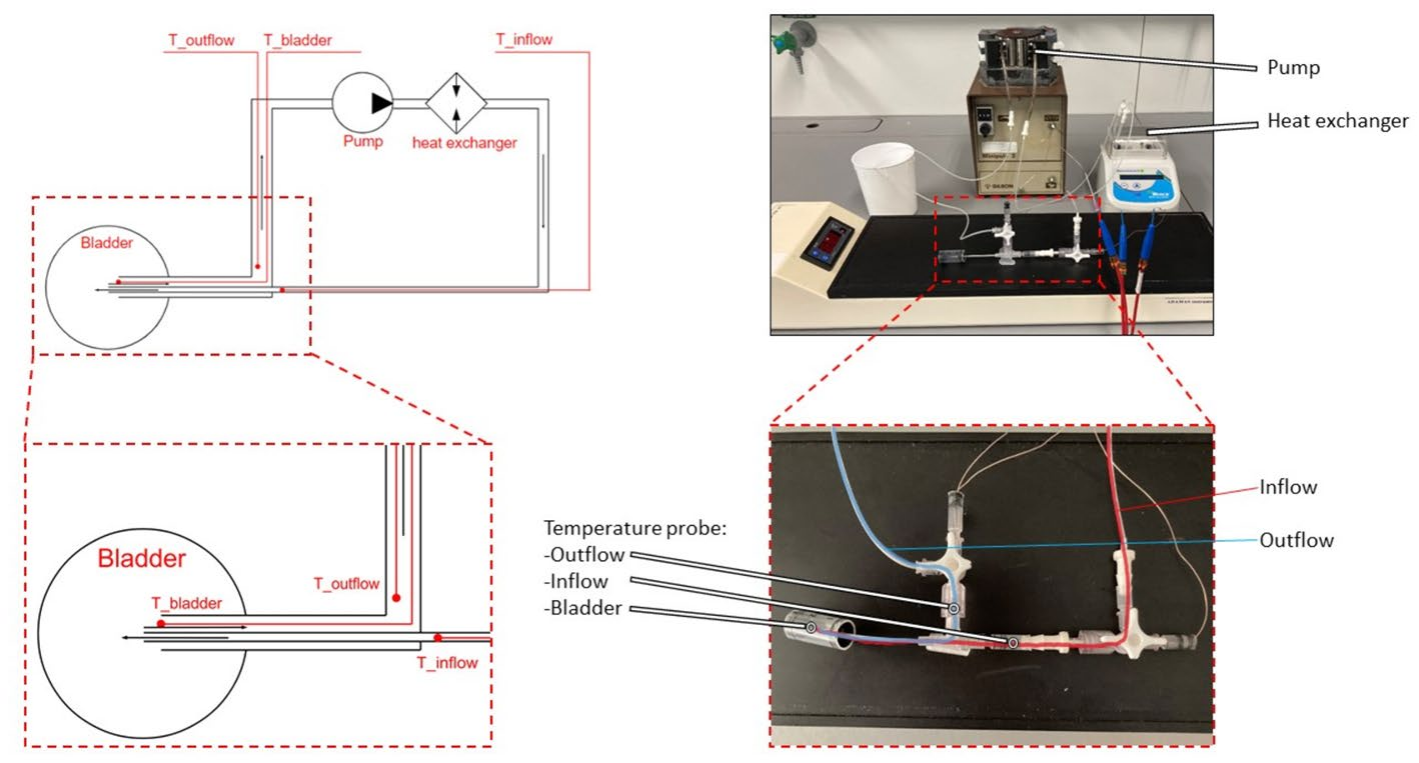
Overview of the HIVEC setup. Top left, schematic overview of setup showing the semi-open circulatory loop, flow direction and location of temperature probes. Bottom left, zoom-in of bladder and double-lumen catheter showing placement of temperature probes in the inflow channel, bladder, and outflow channel. Top Right, overview of setup with the bladder phantom. Bottom right, bladder phantom with the double lumen catheter fully inserted. The liquid inflow trajectory is shown in red, the outflow in blue, and the tips of the temperature probes are marked with white dots.

The setup (Fig. 1) (interactive figure and data) is conceptually similar to the clinical COMBAT BRS system (innoMedicus AG, Cham, Switzerland), where an externally heated chemotherapeutic solution is circulated through a double-lumen catheter that is inserted into the patient’s bladder via the urethra. The setup is further modelled on a previously reported bladder instillation system that was designed to reduce background noise during MRI imaging of rodents^46^. The setup is a semi-open system, in which the flow rate is regulated by a peristaltic pump. From the fluid reservoir, the solution is guided through a heating block. The heated solution is then circulated through the bladder via a double-lumen catheter^47^. The inner tube in the double-lumen catheter forces active inflow, while outflow occurs passively through the catheter’s outer tube (Fig. 1), as we found that forcing an active outflow by a closed-loop system results in the trapping of air in the bladder and flow disruptions in the outflow tubing of the catheter, leading to higher fluctuations of the intravesical temperature. Heat loss through the tubing is minimized by insulating foam. The temperature of the circulating solution can be monitored in the (i) inflow channel, (ii) outflow channel, and (iii) bladder lumen, using thin thermocouples inserted via standard y-branched tubing connectors.

### Technical specifications

A peristaltic pump (Minipuls 2, Gilson, Villiers le Bel, France) was used to circulate the liquid through a semi-open loop of insulated silicone tubing (autoclavable nylon N3A, inner diameter (ID) = 1.00 mm, outer diameter (OD) = 1.78 mm, Smiths Medical, Minneapolis, Minnesota USA). One-way connectors (REF 1050, VBM Medizintechnik GmbH, Sulz am Neckar, Germany) were used to connect the different parts of the circulatory system. The sections of the system between the pump and the bladder, and between the bladder and the pump are referred to as the inflow channel and outflow channel, respectively.

Heating was achieved by passing a section of the silicone tubing, without insulation, through a manually-adjustable laboratory heating block (BSH 200, Benchmark Scientific Inc. Edison, New Jersey USA) with a custom block, machined out of aluminum, that tightly encapsulated 4 loops of tubing (~ 27 cm) to ensure efficient heat transfer between the heating block and the liquid circulating in the tubing. Downstream from the heating block, the silicone tubing was connected to a section of thin intramedic polyethylene tubing (ID = 0.61 mm, OD = 0.28 mm, Becton Dickinson and Company, Franklin Lakes, New Jersey USA). This connection was realized using a cut-off tip and the rubber plunger of a standard 1 mL luer syringe (BD Plastipak, Becton Dickinson S.A., Camino de Valdeoliva, Madrid, Spain). The thin tubing was threaded through a hole pierced in the plunger using a hypodermic needle. The plunger was then installed inside the cut-off syringe, serving as a seal, such that a sufficient length (~ 7 cm) of the thin tubing protruded through the tip of the syringe. Upon inserting into a standard IV catheter (22 G for mice or 17 G for rats, Vasofix Safety IV Catheter with injection port, B. Braun Melsungen AG, Melsungen, Germany), the thin tubing formed the catheter’s inner lumen, with the length of the thin tubing chosen such that it protruded ~ 2 mm beyond the catheter after full insertion, to deliver the heated liquid into the bladder. The lumen of the i.v. catheter and its standard syringe port were connected to silicone tubing (outflow channel), such that after exiting the bladder through the other lumen of the catheter, the liquid was directed to an open-top reservoir and, via more silicone tubing was led through the peristaltic pump, closing the system loop. The tubing system had additional three-way connectors, close to the catheter, to enable thermometry (Fig. 1).

### Thermometry

Three constantan-copper multipoint temperature probes (diameter 0.5 mm, Volenec RD Int, Trebes, Czech Republic) were placed in the inflow channel, bladder lumen and outflow channel, via three-way connectors (in the inflow and outflow channels) and via the outer lumen of the catheter (in the bladder lumen), and plugged into a custom-made thermometry system with an accuracy better than 0.2 °C. Temperature was readable in real-time and logged every second. Temperature corrections were possible by adjusting the temperature of the heating block, while maintaining a fixed flow rate. Temperatures of the skin, external bladder wall and pelvis were measured with a thermal camera (FLIR one Pro, Teledyne FLIR LLC, Wislonville, Oregon, USA). The abdomen of rats was not shaved before treatment.

### Flow rate measurements

The inflow or outflow tract were placed in an Erlenmeyer. From the moment the setup was turned on, a scale was used to assess weight increases on-the-minute, providing a basis for estimating the flow rate by assuming a water density of 1 g per mL.

### Treatment procedures

The outer part of the double lumen catheter was lubricated (Instillagel, Farco-Pharma GmbH, Köln, Germany) and inserted into the bladder phantom or into the bladder via the urethra of female rats. The inner part of the catheter, connected to the inflow channel via a cut-off 1 mL luer syringe (as described above), was then gently inserted into the catheter. Three-way connectors (BD Connecta luer-lok, Becton Dickinson Infusion Therapy AB, Helsingburg, Sweden) were used to introduce the temperature probes at three locations in the system (Fig. 1).

### Bladder phantom

The bladder phantom was constructed by cutting down the barrel of a 5 mL syringe (Becton Dickinson Infusion Therapy AB, Helsingburg, Sweden) to 2 mL. The tube was closed by inserting rubber plungers from two 5 mL syringes on both ends, such that volume adjustment was possible by moving the rubber plungers. The catheter was introduced into the bladder phantom through one of the plungers with a needle from a Vasofix Safety IV Catheter.

### Validation

We validated the setup by performing a one-hour HIVEC instillation in (i) a plastic bladder phantom, with a volume of ~ 1 mL—comparable to that of a rat bladder, (ii) freshly euthanised rats, and (iii) live rats under general anaesthesia. We evaluated the system's ability to reach and maintain a clinically relevant intravesical temperature of 42–43 °C for one hour. With the thermal camera we mapped the heat distribution during the procedure and evaluated whether uniform heating of the bladder wall was achieved. Temperature data were gathered from 4, 8 and 3 independent experiments in the bladder phantom, euthanised and anesthetised rats respectively.

### Animal experiments

All experimental protocols were approved by the Animal Research Institute (ARIA) of the Amsterdam University Medical Centers under project number 17–3746. Experiments were performed in accordance with Dutch legislation on animal experimentation and in compliance with the ARRIVE guidelines.

#### Euthanised rats

Eight female Lewis rats, 6–10 months of age, were euthanised using 100% CO_2_ and treated within two hours after euthanisation. The body temperature after euthanisation was increased by placing animals on a heating plate bench heated to at 37 °C. After 60 min of treatment, the abdomen was opened to inspect the bladder wall for possible perforations or other signs of damage. To assess the temperature of the external bladder wall during treatments, the abdomen of selected animals was opened before the start of the treatment and monitored throughout the entire procedure.

#### Anesthetised rats

Three six-months-old female Lewis rats were anesthetised before treatment using 2% isoflurane in 100% oxygen, after pretreatment with 0.05 mg/kg buprenorphine. The body temperature was maintained throughout the procedures by placing rats on a 37 °C heated mattress bench. After transurethral catheterisation and during HIVEC treatments, the animals were continuously monitored for macroscopic hematuria or blood clots in the catheter and circulating system. During treatment, the lower abdomen of the rats was massaged delicately every five minutes to prevent air bubbles from accumulating in the bladder. After 60 min, the abdomen was opened to monitor for any signs of damage and to measure the bladder wall temperatures with the thermal camera. When treatment was completed, rats were euthanised using 100% CO_2_ while under anesthesia.

### Data availability, analysis and statistics

Statistical analyses were performed using GraphPad Prism version 9. One way ANOVA analysis with Dunnett’s multiple comparisons test was used to compare the temperatures at different locations in the system. Statistical significance was assumed for adjusted *p* values < 0.05. Interactive charts and figures were produced using the FiglinQ platform (https://figlinq.com). All source data and figures can be explored at *Figlinq*.

## Results

### Validation

**Figure 2:**
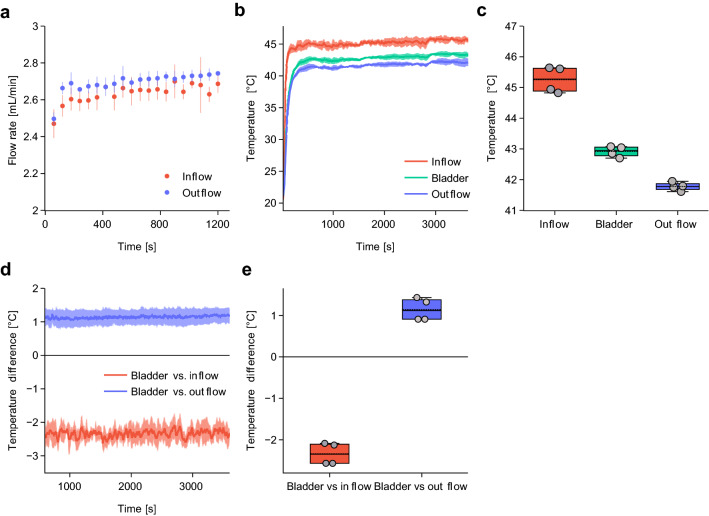
Setup validation using a bladder phantom. (**a**) Flow rates of the inflow and outflow in bladder phantom at fixed pump setting of 2.5 mL/min (n = 3). (**b**) Average temperature kinetics during one hour of HIVEC in the bladder phantom. (**c**) Average temperature during the steady state phase (600–3600 s). (**d**) Temperature difference between the bladder and the inflow/outflow channels during steady-state phase. (**e**) Average temperature difference between the bladder and inflow/outflow channels during steady state phase. All data are mean ± SD. All temperature data were gathered in four independent experiments.

Using the phantom, we observed minimal fluctuations of the liquid flow rate in the inflow- and outflow tubing, demonstrating that stable flow rates in the entire system can be achieved and maintained over extended periods of time (Fig. 2a) (interactive figure and data). We subsequently evaluated the system's ability to reach and maintain a clinically relevant intravesical temperature of 42–43 °C for one hour. We set the flow rate at 2.5 mL/min while adjusting the temperature of the heating block if necessary. Clinical convective systems use an adjustable flow rate up to 200 mL/min^23^. Since the bladder volume of rats (1.0 mL) is much smaller than humans 500 ml we found that changing the flow rate had a significant negative impact on the temperature control. With a flow rate of 2.5 mL/min we were able to ensure rapid and stable heating of the bladder lumen. At lower flow rates we observed significant heat loss in the inflow channel. This resulted in a greater temperature gradient between the inflow and outflow channel and led to instable temperatures inside the bladder. By monitoring the temperature in the inflow channel, the phantom lumen and the outflow channel, as depicted in Fig. 1, we found that the target intra-luminal temperature could be reached within 10 min (Fig. 2b). Temperatures averaged over the subsequent steady-state phase of the treatment were 45.25 °C (SD = 0.37 °C), 42.92 °C (SD = 0.15 °C), 41.77 °C (SD = 0.12 °C) in the inflow channel, phantom lumen and outflow channel, respectively (Fig. 2b,c).

During this phase, we also observed a near-constant temperature difference between the inflow/outflow channel and the phantom lumen (Fig. 2d,e). The average temperature difference between the phantom lumen and the inflow channel was 2.34 °C (*p* < 0.0001), while outflow temperatures were 1.14 °C lower than in the bladder phantom (*p* = 0.002). The variation in temperature difference between the bladder phantom and outflow channel was slightly lower than that between the bladder phantom and the inflow channel (Fig. 2d). Data from all individual treatments and respective differences are shown in Supplementary Figs. S1–S3 (interactive figure and data).

**Figure 3:**
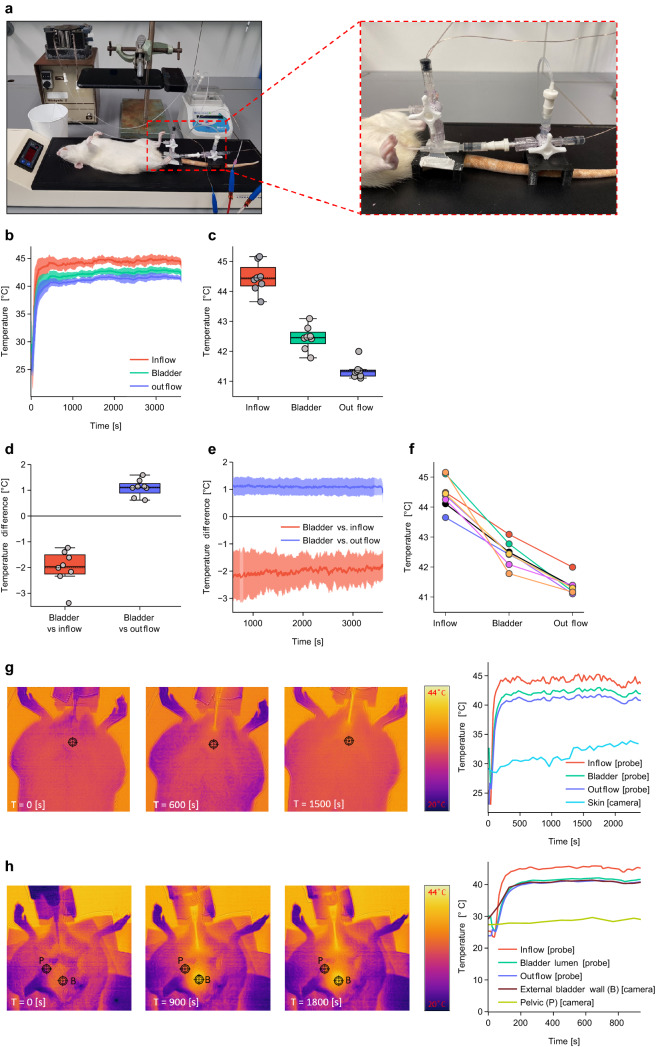
Temperature measurements during HIVEC in euthanised rats. (**a**) Left; overview of setup with an euthanized rat and thermal camera, Right; close up of fully assembled double-lumen catheter inserted into the bladder. (**b**) Average temperature kinetics during the entire treatment. (**c**) Average temperature during the steady state phase of the treatment (600–3600 s). (**d**) Temperature difference between the bladder and inflow/outflow channels during the steady state phase. (**e**) Average temperature difference between the bladder and inflow/outflow channels during steady state phase. (**f**) Connected average bladder, inflow and outflow temperatures of individual treatments during the steady state phase. (**g**) Left; thermal camera image of rat 7 during the procedure. Right; corresponding temperatures during the procedure of rat 7 with skin temperature obtained from the thermal camera. (**h**) Left; thermal camera image of rat 8 with opened abdomen during HIVEC. Right; corresponding temperatures with external bladder wall and pelvic temperatures obtained from the thermal camera. Data are mean ± SD. Temperature data were gathered from 8 independent experiments. Experiments with thermal camera were performed, one representative measurement is shown.

**Figure 4:**
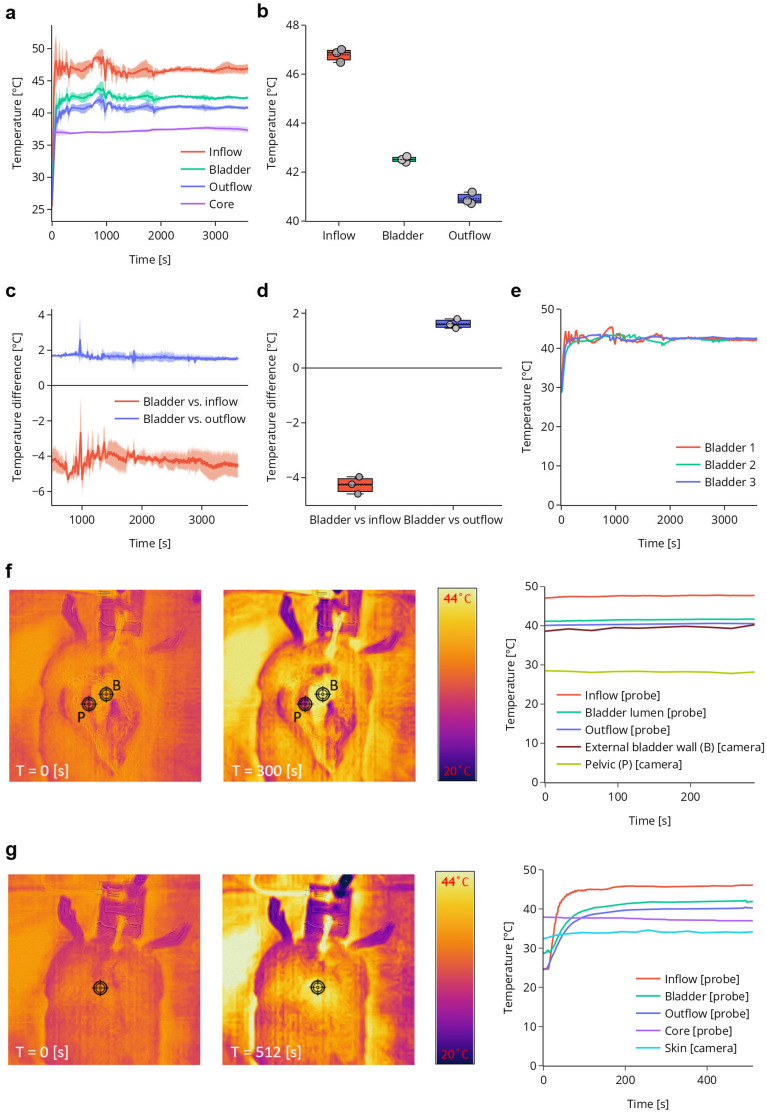
Temperature measurements during HIVEC in live anesthetised rats. (**a**) Average temperature kinetics during the entire treatment. (**b**) Average temperatures during the steady-state phase of the treatment. (**c**) Temperature differences between the bladder lumen and inflow/outflow channels during the steady state phase. (**d**) Average temperature difference between bladder lumen and inflow/outflow channels during the steady-state phase of treatment. (**e**) Individual bladder lumen temperature kinetics during the entire procedure. (**f**) Left; thermal camera image from rat 2 at the start of the procedure and after 500 s, Right; corresponding core and skin temperatures, measured with the thermal camera. (**g**) Left; thermal camera image at start and after 300 s, with opened abdomen, exposing the pelvis and bladder wall. Right; corresponding (other) temperatures in the system. Data are mean ± SD. Temperature measurements were gathered from 3 independent experiments. Thermal camera imaging measurements were performed in all rats and showed consistent heating kinetics between individual experiments.

We then proceeded to validate our setup in euthanised (Fig. 3) (interactive figure and data) and live, anaesthetized (Fig. 4) (interactive figure and data) rats. We observed similar heating kinetics as when using the bladder phantom (Fig. 3b,c), with adequate temperature control and consistent difference between the inflow, the lumen and the outflow. The average luminal bladder temperature during the stable phase of the treatment was 42.45 °C (SD = 0.37 °C) and 42.52 °C (SD = 0.09 °C) in euthanised (Fig. 3c) and anaesthetized (Fig. 4b) rats, respectively. The temperature difference between the bladder and the inflow/outflow channels in euthanised rats was comparable to that measured in the bladder phantom, showing lower variation between the lumen and the outflow (mean ΔT = 1.09 °C, SD = 0.30 °C, *p* < 0.0001) than between the bladder and the inflow (mean ΔT = 2.01 °C, SD = 0.63 °C, *p* < 0.0001) (Figs. 3b,d,e). Although the differences between luminal vs inflow and luminal vs outflow temperatures were consistent between measurements in euthanised rats, the highest luminal temperatures did not always correspond with the highest temperatures in the inflow or outflow channels (Fig. 3f).

During HIVEC in anaesthetized rats, we initially observed a greater temperature difference between the inflow, the bladder lumen and the outflow, which nevertheless did not affect the mean luminal temperature (Fig. 4d-f). The individual bladder temperatures showed a trend of decreasing temperature variation in consecutive measurements (Fig. 4d, S3). Maintaining stable temperature in anaesthetized rats 1 and 2 required some manual correction of the heating block settings during treatment, but the results demonstrate that such adjustments are feasible and effective.

To evaluate whether uniform and local heating of the bladder wall was achieved, we used a thermal camera and mapped heat distribution during the procedure. Imaging of the animal skin surface showed a small temperature increase during the first phase of the treatment (Fig. 3f and Fig. 4g). During the first 8 min of the treatment in anaesthetized rat 2, its skin surface temperature increased from 32.36 °C to 34.15 °C, while the luminal bladder temperature reached a stable temperature of 41.83 °C within this time (Fig. 4f). Importantly, after opening the abdominal and pelvic cavity during the procedure we observed uniform heating of the bladder (Fig. 3h), with a minimal temperature increase of the surrounding tissue. The average luminal bladder temperature during treatment in euthanised rats was 42.45 °C (SD = 0.37 °C), corresponding to an external bladder wall temperature of 41.15 °C (Fig. 3g). A temperature probe placed non-invasively in the oesophagus of anaesthetized rats showed no increase in the body temperature (Fig. 4a). We also did not observe any signs of haematuria or blood clots after catheterization or during the procedure.

## Discussion

An optimal HIVEC setup should provide a sufficiently high intraluminal temperature that can be maintained over extended periods of time. Compared to published temperature data from clinical HIVEC trials, our system is able to reach and maintain relevant bladder temperatures with low variability within each instillation and between different instillations^48^. Furthermore, clinically recorded median temperatures are generally lower than the temperatures that we were regularly able to reach in our experiments^48^, suggesting that our setup is capable of a well-controlled thermal dose. The small variability between measurements also enables treatment at different thermal doses. Importantly, the temperature increase remained mostly confined to the bladder, which is critical for protection of surrounding organs.

Uneven heat distribution inside the bladder can lead to the so-called cold- and hot spots. These can occur due to electric field heterogeneity in capacitive heating techniques or due to the formation of air bubbles in the bladder during the treatment^49,50^. Hot spots can lead to heating of surrounding structures within the range of the electric field, such as the rectum and vagina, while simultaneously creating uneven heating of the bladder wall, depending on the position relative to RF source^36,37,39^. Measurements using a thermal-vision camera showed homogenous heating of the external bladder wall (Figs. 3h, 4g). In some in vivo studies focusing on the thermal distribution during HIVEC in larger animals (e.g. sheep or pig), there was a need to surgically place thermal probes in the pelvis and in different layers of the bladder wall^35,38^. Due to the thin bladder wall of rats (100–120 µm thickness of the detrusor muscle)^51^, this was not possible in our setup, but also not necessary in view of the small bladder volume. One concern in HIVEC is formation of air bubbles, which can disturb the flow patterns and cause uneven heat distribution. In our system, we believe this can be minimized by regular abdominal massage of the pelvic region. Finally, as the system will ultimately be used in heterotopic tumour models, accurate thermometry will still be needed to evaluate the effect of bladder tumours on heat distribution.

When treating live, anesthetised rats, the temperature variability was larger than what we expected based on the prior experiments with bladder phantom and euthanised rats (Figs. 2b, 3b, 4a). This can be explained by the fact that treatment of live rats was executed in a flow cabinet with a lower ambient temperature, leading to an increased temperature loss of the tubing system to the environment. This had to be compensated for by increasing the inflow temperature, and caused a larger difference between the bladder and inflow channels (Fig. 4d). The lower ambient temperature can also explain the decreased bladder wall temperature measured in live anesthetised rats, as compared to euthanised rats (Figs. 3h, 4g). Importantly, however, the intraluminal thermal dose was not affected by these external fluctuations, underscoring the robustness of the system.

Since in vivo HIVEC treatment schedules usually consist of a series of repeated instillations, and are generally applied to groups of animals, it is important to ensure a minimally invasive, stable and scalable setup. Although we did not observe hematuria or clot formation during treatment, a possibility remains of urethral or bladder mucosal damage that could lead to dysuria and animal discomfort, especially upon repeated application. Further experiments are needed to explore such effects. The study by Haveman et al. showed, however, that the effects of a similar procedure on rat bladder volume and blood urea nitrogen levels normalize within days^36^. To support experiments with larger numbers of animals, the complexity and invasiveness of the setup can be lowered even further. Although we measured the temperature at three different locations, the outflow temperature showed a relatively constant deviation from the intraluminal temperature in all experiments, suggesting that it can serve as a surrogate for bladder temperature. Eliminating the intraluminal sensor will facilitate the application procedures, reduce the impact on the urinary tract of the treated animals, and may make the setup compatible with smaller rodents (e.g. mice), where the dimensions of the bladder and urinary tract complicate temperature monitoring in the bladder. In fact, we found that when equipped with a thinner catheter (22 gauge), HIVEC instillations are indeed feasible in mice. In addition, our approach can accommodate simultaneous treatment of multiple animals by splitting the inflow channel (and adjusting the flow rate/heating block temperature accordingly), or by using extra heating blocks/circulatory loops attached to a single pump, or even multiple pumps. To further optimize the setup for larger-scale experiments, automated temperature control could be implemented based solely on the outflow temperature.

In summary, this study presents a simple, scalable, reliable, and cost-effective setup for HIVEC treatments in rodents. The system achieves rapid and stable local heating of the bladder lumen and bladder wall, while continuous, multi-point monitoring allows for swift temperature adjustments, if required. The setup can already be used to study hyperthermia-based treatment combinations in vivo, while further improvements can render it even less invasive and suitable for repeated, larger scale experiments, in both rats and mice.

## Supplementary Information

Supplementary Information.

## Data Availability

All source data and figures can be explored at Figlinq.

## References

[1] Bray F (2018). Global cancer statistics 2018: GLOBOCAN estimates of incidence and mortality worldwide for 36 cancers in 185 countries. CA Cancer J. Clin..

[2] EAU Guidelines: Non-muscle-invasive Bladder Cancer | Uroweb. https://uroweb.org/guideline/non-muscle-invasive-bladder-cancer/#11.

[3] Rj S (2021). European Association of Urology (EAU) Prognostic Factor Risk Groups for Non-muscle-invasive Bladder Cancer (NMIBC) incorporating the WHO 2004/2016 and WHO 1973 classification systems for grade: An update from the EAU NMIBC guidelines panel. Eur. Urol..

[4] Nederlandse Kankerregistratie (NKR), IKNL. www.iknl.nl/nkr-cijfers.

[5] Porter MP, Gore JL, Wright JL (2011). Hospital volume and 90-day mortality risk after radical cystectomy: A population-based cohort study. World J. Urol..

[6] Hautmann RE, de Petriconi RC, Volkmer BG (2010). Lessons learned from 1,000 neobladders: The 90-day complication rate. J. Urol..

[7] Yang LS (2016). A systematic review and meta-analysis of quality of life outcomes after radical cystectomy for bladder cancer. Surg. Oncol..

[8] Stauffer PR, van Rhoon GC (2016). Overview of bladder heating technology: Matching capabilities with clinical requirements. Int. J. Hyperth..

[9] Sousa A (2014). A clinical trial of neoadjuvant hyperthermic intravesical chemotherapy (HIVEC) for treating intermediate and high-risk non-muscle invasive bladder cancer. Int. J. Hyperth..

[10] van Rhoon GC, Franckena M, ten Hagen TLM (2020). A moderate thermal dose is sufficient for effective free and TSL based thermochemotherapy. Adv. Drug Deliv. Rev..

[11] Milla P (2014). Intravesical thermo-chemotherapy based on conductive heat: A first pharmacokinetic study with mitomycin C in superficial transitional cell carcinoma patients. Cancer Chemother. Pharmacol..

[12] Mikhail AS (2017). Lyso-thermosensitive liposomal doxorubicin for treatment of bladder cancer. Int. J. Hyperth..

[13] Richter K, Haslbeck M, Buchner J (2010). The heat shock response: Life on the verge of death. Mol. Cell.

[14] Scutigliani EM, Liang Y, Crezee H, Kanaar R, Krawczyk PM (2021). Modulating the heat stress response to improve hyperthermia-based anticancer treatments. Cancers.

[15] Oei AL, Vriend LEM, Crezee J, Franken NAP, Krawczyk PM (2015). Effects of hyperthermia on DNA repair pathways: One treatment to inhibit them all. Radiat. Oncol..

[16] Gabano E, Colangelo D, Ghezzi AR, Osella D (2008). The influence of temperature on antiproliferative effects, cellular uptake and DNA platination of the clinically employed Pt(II)-drugs. J. Inorg. Biochem..

[17] Ohtsubo T (1997). Enhancement of cisplatin sensitivity and platinum uptake by 40°C hyperthermia in resistant cells. Cancer Lett..

[18] Wallner KE, Li GC, DeGregorio MW (1986). Hyperthermic potentiation of cis-diamminedichloroplatinum(ii) cytotoxicity in chinese hamster ovary cells resistant to the drug. Cancer Res..

[19] van der Heijden AG, Dewhirst MW (2016). Effects of hyperthermia in neutralising mechanisms of drug resistance in non-muscle-invasive bladder cancer. Int. J. Hyperth..

[20] van der Heijden AG, Verhaegh G, Jansen CFJ, Schalken JA, Witjes JA (2005). Effect of hyperthermia on the cytotoxicity of 4 chemotherapeutic agents currently used for the treatment of transitional cell carcinoma of the bladder: An in vitro study. J. Urol..

[21] van der Heijden AG (2004). The effect of hyperthermia on mitomycin-C induced cytotoxicity in four human bladder cancer cell lines. Eur. Urol..

[22] Helderman RFCPA (2020). The Temperature-dependent effectiveness of platinum-based drugs mitomycin-C and 5-FU during hyperthermic intraperitoneal chemotherapy (HIPEC) in colorectal cancer cell lines. Cells.

[23] Tan WP (2020). Safety and efficacy of intravesical chemotherapy and hyperthermia in the bladder: Results of a porcine study. Int. J. Hyperth..

[24] Longo FW, Tomashefsky P, Rivin BD, Tannenbaum M (1983). Interaction of ultrasonic hyperthermia with two alkylating agents in a murine bladder tumor. Cancer Res..

[25] Amano T, Kumini K, Nakashima K, Uchibayashi T, Hisazumi H (1990). A combined therapy of hyperthermia and tumor necrosis factor for nude mice bearing KK-47 bladder cancer. J. Urol..

[26] Stapf M, Teichgräber U, Hilger I (2017). Methotrexate-coupled nanoparticles and magnetic nanochemothermia for the relapse-free treatment of T24 bladder tumors. Int. J. Nanomed..

[27] Arends TJH (2016). Results of a randomised controlled trial comparing intravesical chemohyperthermia with mitomycin C versus bacillus calmette-guérin for adjuvant treatment of patients with intermediate- and high-risk non-muscle-invasive bladder cancer. Eur. Urol..

[28] De Jong JJ, Hendricksen K, Rosier M, Mostafid H, Boormans JL (2018). Hyperthermic intravesical chemotherapy for BCG unresponsive non-muscle invasive bladder cancer patients. Bladder Cancer.

[29] Liu K (2020). Thermal intravesical chemotherapy reduce recurrence rate for non-muscle invasive bladder cancer patients: A meta-analysis. Front. Oncol..

[30] Brummelhuis ISG (2021). Long-term experience with radiofrequency-induced hyperthermia combined with intravesical chemotherapy for non-muscle invasive bladder cancer. Cancers.

[31] Ruan JL (2019). Mouse models of muscle-invasive bladder cancer: Key considerations for clinical translation based on molecular subtypes. Eur. Urol. Oncol..

[32] Arentsen HC, Hendricksen K, Oosterwijk E, Witjes JA (2009). Experimental rat bladder urothelial cell carcinoma models. World J. Urol..

[33] Oršolić N, Odeh D, Jembrek MJ, Knežević J, Kučan D (2020). Interactions between cisplatin and quercetin at physiological and hyperthermic conditions on cancer cells in vitro and in vivo. Molecules.

[34] Uçar M (2016). The effect of thermochemotherapy with mitomycin C on normal bladder urothelium, an experimental study. Int. Urol. Nephrol..

[35] van Valenberg FJP (2021). DPPG2-based thermosensitive liposomes with encapsulated doxorubicin combined with hyperthermia lead to higher doxorubicin concentrations in the bladder compared to conventional application in pigs: A rationale for the treatment of muscle-invasive bladder cancer. Int. J. Nanomed..

[36] Haveman J, Smals OAG, Rodermond HM (2003). Effects of hyperthermia on the rat bladder: A pre-clinical a study on thermometry and functional damage after treatment. Int. J. Hyperth..

[37] Salahi S (2012). Miniature microwave applicator for murine bladder hyperthermia studies. Int. J. Hyperth..

[38] Rath-Wolfson L, Moskovitz B, Dekel Y, Kugel V, Koren R (2003). Combined intravesical hyperthermia and mitomycin chemotherapy: A preliminary in vivo study. Int. J. Exp. Pathol..

[39] Oliveira TR (2013). Magnetic fluid hyperthermia for bladder cancer: A preclinical dosimetry study. Int. J. Hyperth..

[40] Yang X (2017). The Antineoplastic activity of photothermal ablative therapy with targeted gold nanorods in an orthotopic urinary bladder cancer model. Bladder Cancer.

[41] Hood RL (2013). Spatially controlled photothermal heating of bladder tissue through single-walled carbon nanohorns delivered with a fiberoptic microneedle device. Lasers Med. Sci..

[42] Zhu L (2019). Feasibility and safety assessment of magnetic resonance-guided high-intensity focused ultrasound (MRgHIFU)-mediated mild hyperthermia in pelvic targets evaluated using an in vivo porcine model. Int. J. Hyperth..

[43] Ba M (2019). Development of a high-precision bladder hyperthermic intracavitary chemotherapy device for bladder cancer and pharmacokinetic study. BMC Urol..

[44] Oliveira TR (2013). Preclinical dosimetry of magnetic fluid hyperthermia for bladder cancer. Proc. SPIE Int. Soc. Opt. Eng..

[45] Priester MI, Curto S, van Rhoon GC, Ten Hagen TLM (2021). External basic hyperthermia devices for preclinical studies in small animals. Cancers.

[46] Haney CR (2006). Reduction of image artifacts in mice by bladder flushing with a novel double-lumen urethral catheter. Mol. Imag..

[47] Reis LO (2011). Anatomical features of the urethra and urinary bladder catheterization in female mice and rats. An essential translational tool. Acta Cir. Bras..

[48] Geijsen ED (2015). Combining mitomycin C and regional 70 MHz Hyperthermia in patients with nonmuscle invasive bladder cancer: A pilot study. J. Urol..

[49] Schooneveldt G (2018). The effect of air pockets in the urinary bladder on the temperature distribution during loco-regional hyperthermia treatment of bladder cancer patients. Int. J. Hyperth..

[50] Brummelhuis I, Crezee J, Witjes A (2021). Pd63–02 the thermal dose during radiofrequency-induced hyperthermia combined with intravesical chemotherapy appears to have no significant effect on non-muscle invasive bladder cancer outcome. J. Urol..

[51] Gabella G, Uvelius B (1990). Urinary bladder of rat: Fine structure of normal and hypertrophic musculature. Cell Tissue Res..

