# Supplementary material for: A scalable hyperthermic intravesical chemotherapy (HIVEC) setup for rat models of bladder cancer
Source: Sci Rep. 2022 Apr 29;12:7017. doi: 10.1038/s41598-022-11016-y (PMC9054747; doi:10.1038/s41598-022-11016-y)
Supplement: Supplementary file 1 — Supplementary Information. [file 41598_2022_11016_MOESM1_ESM.pdf]

A Scalable Hyperthermic Intravesical Chemotherapy (HIVEC) Setup for Rat Models of Bladder Cancer

van Hattum et al. Figure S1

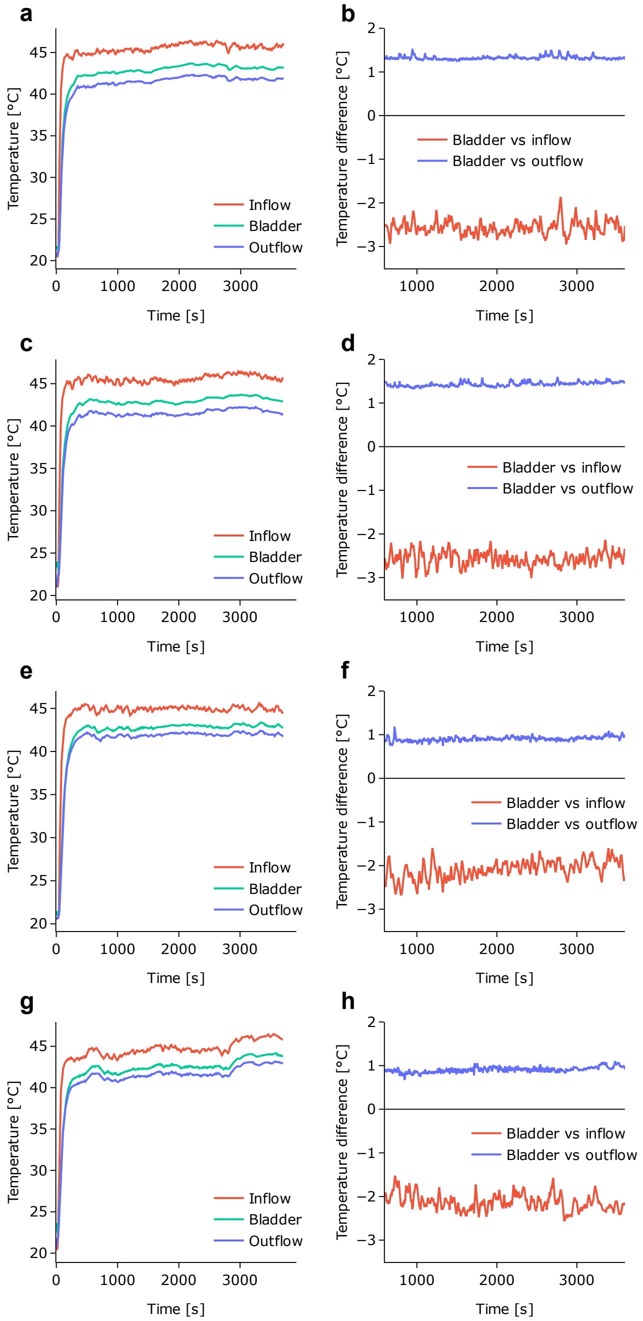

van Hattum et al. Figure S2

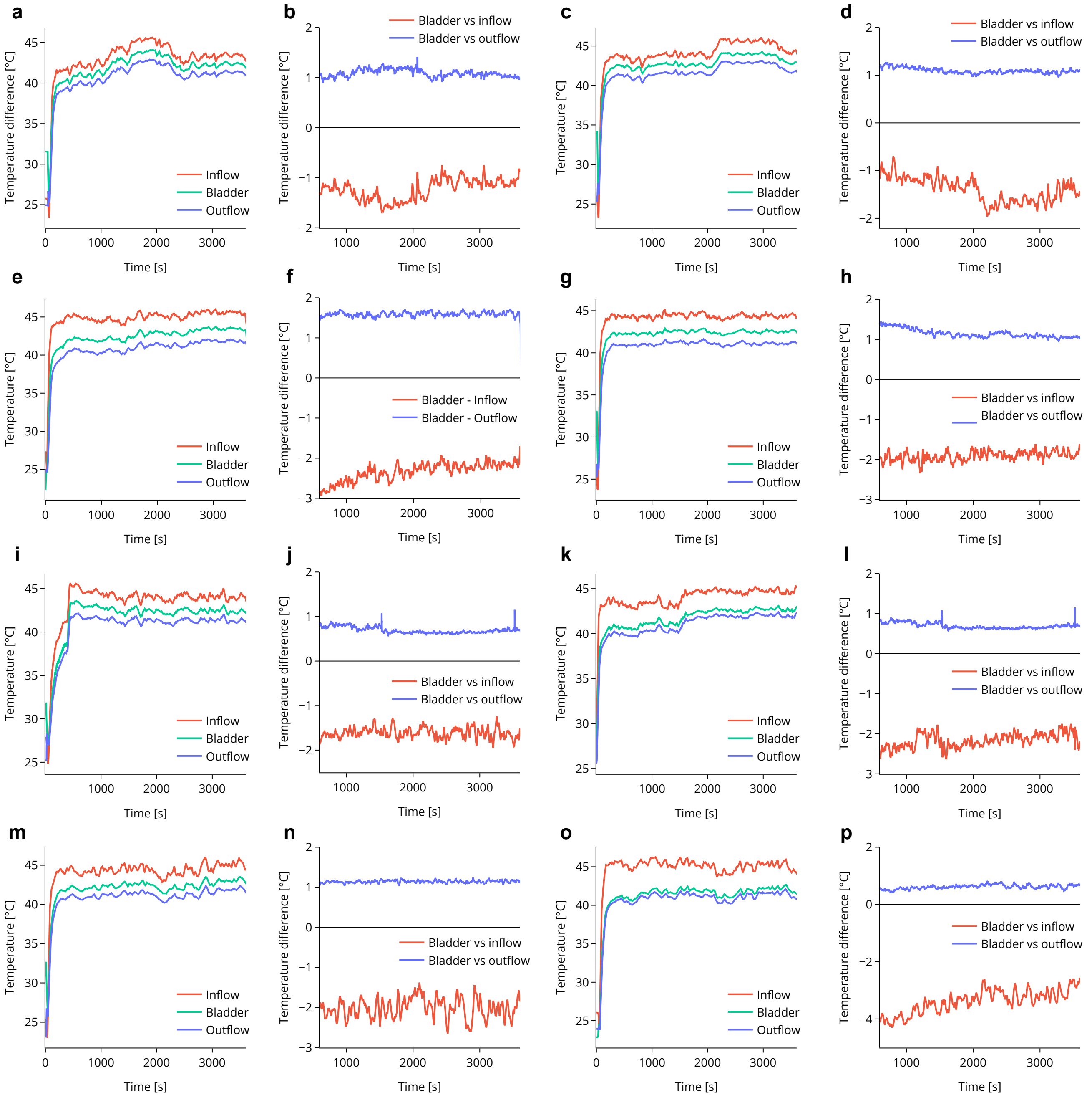

van Hattum et al. Figure S3

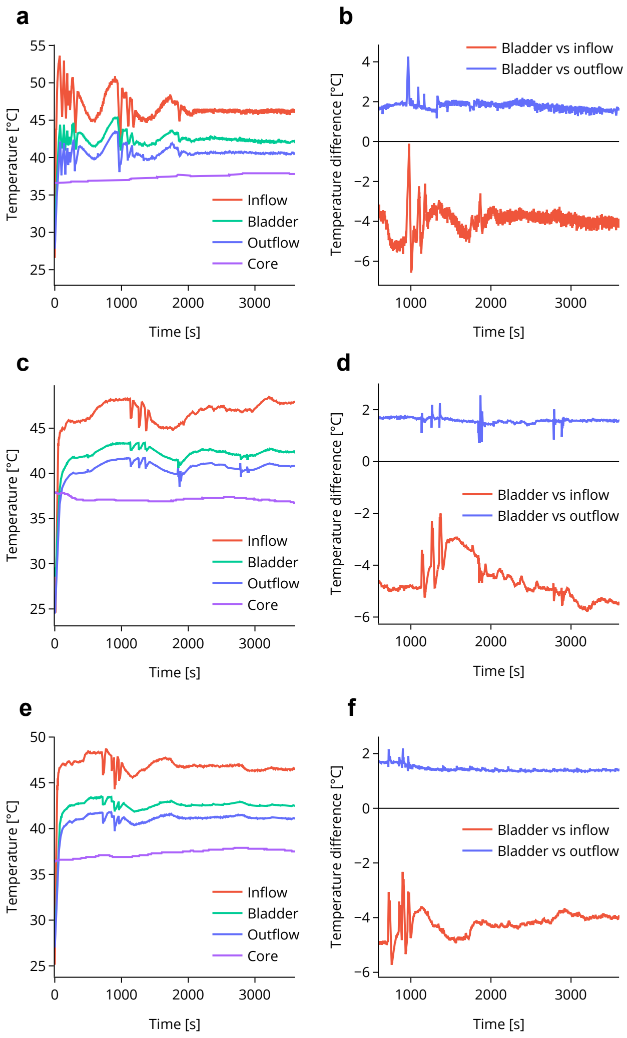

Supplementary figure legends

Figure S1. Individual temperature measurements during HIVEC in the bladder phantom. Each row represents an individual measurement, with left-hand-side panels showing the temperature kinetics throughout the procedure, and right-hand-side panels the corresponding temperature difference between the bladder and the inflow/outflow channel.

Figure S2. Individual temperature measurements during HIVEC in euthanised rats. Each row represents two individual measurements, with left-hand-side panels showing the temperature kinetics throughout the procedure, and right-hand-side panels the corresponding temperature difference between the bladder and the inflow/outflow channel.

Figure S3. Individual temperature measurements during HIVEC in anesthetised live rats. Each row represents an individual measurement, with left-hand-side panels showing the temperature kinetics throughout the procedure, and right-hand-side panels the corresponding temperature difference between the bladder and inflow/outflow channel.
